# *Salmonella enterica* Serovars Enteritidis Infection Alters the Indigenous Microbiota Diversity in Young Layer Chicks

**DOI:** 10.3389/fvets.2015.00061

**Published:** 2015-11-23

**Authors:** Khin K. Z. Mon, Perot Saelao, Michelle M. Halstead, Ganrea Chanthavixay, Huai-Chen Chang, Lydia Garas, Elizabeth A. Maga, Huaijun Zhou

**Affiliations:** ^1^Department of Animal Science, University of California Davis, Davis, CA, USA

**Keywords:** *Salmonella* Enteritidis, chickens, gut microbiota, 16S rRNA, MHC haplotypes

## Abstract

Avian gastrointestinal (GI) tracts are highly populated with a diverse array of microorganisms that share a symbiotic relationship with their hosts and contribute to the overall health and disease state of the intestinal tract. The microbiome of the young chick is easily prone to alteration in its composition by both exogenous and endogenous factors, especially during the early posthatch period. The genetic background of the host and exposure to pathogens can impact the diversity of the microbial profile that consequently contributes to the disease progression in the host. The objective of this study was to profile the composition and structure of the gut microbiota in young chickens from two genetically distinct highly inbred lines. Furthermore, the effect of the *Salmonella* Enteritidis infection on altering the composition makeup of the chicken microbiome was evaluated through the 16S rRNA gene sequencing analysis. One-day-old layer chicks were challenged with *S*. Enteritidis and the host cecal microbiota profile as well as the degree of susceptibility to *Salmonella* infection was examined at 2 and 7 days post infection. Our result indicated that host genotype had a limited effect on resistance to *S*. Enteritidis infection. Alpha diversity, beta diversity, and overall microbiota composition were analyzed for four factors: host genotype, age, treatment, and postinfection time points. *S*. Enteritidis infection in young chicks was found to significantly reduce the overall diversity of the microbiota population with expansion of *Enterobacteriaceae* family. These changes indicated that *Salmonella* colonization in the GI tract of the chickens has a direct effect on altering the natural development of the GI microbiota. The impact of *S*. Enteritidis infection on microbial communities was also more substantial in the late stage of infection. Significant inverse correlation between *Enterobacteriaceae* and *Lachnospiraceae* family in both non-infected and infected groups, suggested possible antagonistic interaction between members of these two taxa, which could potentially influences the overall microbial population in the gut. Our results also revealed that genetic difference between two lines had minimal effect on the establishment of microbiota population. Overall, this study provided preliminary insights into the contributing role of *S*. Enteritidis in influencing the overall makeup of chicken’s gut microbiota.

## Introduction

The avian gastrointestinal (GI) tract is home to complex and diverse bacterial populations that provide many beneficial functions to host, which includes conferring colonization resistance against the invading pathogenic microorganisms. Development of the GI microbiota in chickens occurs immediately after hatching and is influenced by both genetic and external factors like diet and environment (1). Unlike other animals, a newly hatched chick does not have acquired healthy maternal microbiota as they are housed separately from the adult hens immediately after hatch in commercial production (2). Therefore, the GI tract of newly hatched chickens is usually sterile and presents an empty ecological niche that provides easy access for the pathogen to colonize with limited restriction (2). This factor alone makes young chickens highly susceptible to enteric bacterial infections, such as *Salmonella*, which can result in different degrees of disease spectrum from a subclinical carrier state to a high mortality rate depending on the infecting bacterial serovar and host’s susceptibility.

*Salmonella enterica* subsp. *enterica* serovar Enteritidis is a zoonotic enteric pathogen that is most frequently associated with diarrheal disease in humans while chickens serve as asymptotic carrier (3). Consumption of contaminated eggs produced by infected layer hens is one of the leading causes of *Salmonella* food poisoning in humans (4). In chickens, *S*. Enteritidis can be easily transmitted horizontally via the fecal–oral route as well as vertically via the reproductive tract, which can contaminate the egg (5). Additionally, chickens can also harbor *S*. Enteritidis asymptomatically and persist throughout their lifespan, which makes the identification of infected chickens and the eradication of the pathogen much more challenging. Young chickens can be exposed to *S*. Enteritidis through numerous external sources like contaminated feed or environment. The sterile GI tract of the newly hatched chickens also provides ample opportunities for a pathogenic organism like *S*. Enteritidis to firmly establish its own niche in the gut as early colonizer and potentially further impact the development of the gut microbiota during the disease state. Early exposure to *Salmonella* in young chick could result in two potential alternative outcomes: high mortality rate or persistence of infection in surviving chickens (6). Prolonged persistent infection with *S*. Enteritidis in the GI tract of chickens throughout their lifespan could alter the development of gut microbiota and have detrimental effect on the overall gut health of the chicken host.

The impact of genetic background on the composition of chicken gut microbiota has been mostly investigated in broilers due to the association of intestinal microbiota with performance of broiler chickens in terms of feed conversion efficiency (7–11). Studies in broiler chickens have indeed shown evidence that host genotype had significant impact on shaping the composition of the gut microbiota (7, 9, 11). Few studies had explored the relationship between the host genotype and its influence on microbiota composition in layer chickens, especially related to disease resistance. The host genetic background plays an important role in the resistance and susceptibility to *Salmonella* infection (12). Several studies have reported that many genes have been found to be associated with *Salmonella* resistance in the chicken (6, 13). One of the key candidate genes, known as major histocompatibility complex (MHC), plays an important role in disease resistance in the chicken (13–20). University of California, Davis (UCD) maintains a number of congenic layer lines differing in MHC B-complex haplotypes. A study by Cotter et al. had previously examined the association of B-complex immunity to *S*. Enteritidis using 12 congenic lines from UCD, differing in various B-complex haplotypes (13). Results from the study had suggested that chickens from UCD254 (B^15^/B^15^) were more susceptible to *Salmonella* infection compared to other lines in term of mortality and morbidity (13). However, underlying mechanism associated with susceptibility to *Salmonella* remains to be elucidated. As microbiota is a significant contributor to disease resistance, two highly inbred line UCD254 (B^15^/B^15^) and UCD077 (B^15^/B^16^) at UCD were used to examine MHC effect on microbial community in chicken intestinal gut.

The main objective of this study was to examine the impact of host genetic background on influencing early establishment of microbiota in combination with *S*. Enteritidis infection to determine *S*. Enteritidis-associated alteration in gut microbiota.

## Materials and Methods

### Experimental Animals

Two genetically distinct, highly inbred layer chickens from line UCD077 and UCD254 were obtained on the day of hatch from UCD’s poultry farm. A cloaca swab was performed to ensure all birds were *Salmonella*-free. The chickens were then transferred and housed in the temperature-controlled chambers with *ad libitum* access to water and commercial feed without antibiotic treatment. At 1 day of age, chickens were orally inoculated with 1 × 10^8^ c.f.u of *S*. Enteritidis TN2 nalidixic acid-resistant strain (kindly provided by Dr. Andreas Baumler) or PBS for the non-infected birds. Dosage of *S*. Enteritidis was confirmed by serial dilution plating of the inoculum. A total of three replicate trials were conducted. For the duration of the trials, all non-infected chickens were housed together in the concrete floor pen with fresh, wood shaving for bedding material inside the environmental chamber. The infected group of chickens was housed separately in another chamber with the same environmental conditions as the control chamber. At 2 and 7 days postinoculation (DPI), chickens were euthanized by the carbon dioxide asphyxiation to collect spleen and cecal content for further analysis. Similarly, the organs from the same-age counterpart in non-infected group of 3 days old (3 D) and 8 days old (8 D) were also collected. All animal experiments were performed according to the guidelines approved by the Institutional Animal Care and Use Committee at the UCD.

### Enumeration of Bacteria in Spleen and Cecal Content

Viable counts of *S*. Enteritidis were recovered from one of the ceca pouches by squeezing its contents into 10 ml PBS and placing immediately on ice after the collection. The second pouch of ceca was collected on ice and frozen at −20°C for the DNA extraction. The weight of the cecum content was measured prior to spreading serial 10-fold dilutions on Xylose Lysine Tergitol-4 (XLT4) selective agar plates containing tetracycline. Similarly, half of the spleen was weighted and homogenized in 1 ml PBS by using the black rubber end of the sterile plunger from the 2 ml syringe before plating. The plates were then incubated at 37°C for 24 h. Counts of *S*. Enteritidis were log transformed and expressed as log_10_ CFU per gram of the cecal content for further statistical analysis.

### DNA Extraction and PCR Amplification of 16S rRNA Gene Sequences

Approximately 150 mg of total cecal content was used for DNA isolation by Zymo fecal DNA miniprep (Zymo Research, Irvine, CA, USA) in accordance with the manufacturer’s instructions. In brief, bead-beating step was performed using the Bullet Blender Storm 24 (Next Advance Inc., Averill Park, NY, USA) for 5 min at maximum speed setting. Concentration and purity of the extracted DNA was measured on the NanoDrop ND-2000C spectrophotometer (ThermoScientific Inc., USA). All extracted DNA samples were stored at −20°C until further analysis. PCR amplification was performed with F515 (5′NNNNNNNN**GT**GTGCCAGCMGCCGCGGTAA3′) and R806 (5′GGACTACHVGGGTWTCTAAT3′) primers targeting the V4 segment of the bacterial 16S rRNA gene where the forward primer was modified to contain the linker region (GT) for sequencing on the Illumina MiSeq platform and a unique 8 bp barcode sequence (*N*) for each sample (21). PCR conditions were set at initial denaturation for 94°C for 3 min; followed by 35 cycles of 94°C for 45 sec, 50°C for 1 min, 72°C for 1 min 30 s with final extension step at 72°C for 10 min. The PCR reaction contained 12.5 μl 2× GoTaq Green Master Mix (Promega, Madison, WI, USA), 9.5 μl nuclease-free water, 0.5 μl forward and reverse primers, and 2.0 μl DNA. All samples were amplified in triplicate and combined after PCR for the purification. The PCR products were inspected on a 1% agarose gel stained with SYBR safe (Life Technologies, CA, USA) and stored at −20°C. The PCR amplicons were purified with QIAquick PCR Purification kit (Qiagen, Valencia, CA, USA) according to the manufacturer’s instruction. The pooled amplicons were then submitted to the University of California, Davis Genome Center, DNA Technology Core Facility for generating 250 paired-end reads on the Illumina MiSeq sequencing platform.

### 16S rRNA Sequence Data Processing

The Quantitative Insights into Microbial Ecology (QIIME) version 1.9.1 was used to analyze the sequencing data generated from three replicate trials samples (Table 1). Raw data were demultiplexed, and quality filtered with QIIME default settings (22). The 250-bp reads were truncated at any site of more than three sequential bases receiving a quality score <Q10 and any read containing ambiguous base calls or barcode/primer errors were discarded as were reads with <75% (of total read length) consecutive high-quality base calls. Similar sequences were clustered together into the operational taxonomic units (OTUs) at 97% identity using QIIME open reference OTU picking against the Greengenes 16S rRNA database (version 13_8) (23).

**Table 1:** **Summary of number of chickens used in each of the replicate trials for 16S rRNA gene sequencing analysis**.

Trial	Line	Non-infected (3 D)	*S*. Enteritidis-infected (2 DPI)	Non-infected (8 D)	*S*. Enteritidis-infected (7 DPI)
1	UCD077	4	11	0	0
	UCD254	4	21	3	11

2	UCD077	5	8	4	5
	UCD254	5	6	5	5

3	UCD077	5	8	5	5
	UCD254	5	10	5	8

*3 D, 3 days old; 8 D, 8 days old; 2 DPI, 2 days post infection; 7 DPI, 7 days post infection*.

### Microbiota Diversity Analysis

Both alpha and beta diversity metrics were used to analyze microbiome composition. Alpha diversity metrics analysis includes Chao1 index (richness estimate), Shannon’s diversity, and Simpson’s diversity index. Chao1 richness estimates the total number of species present in the community. The difference between the Shannon and Simpson indices is that the weights of abundant species are accounted differently. Both the abundance and evenness in distribution of species present in the community is included in Shannon index analysis, while only the abundance of species is considered in Simpson indices (24). Microbial community dominated by a few species is considered to exhibit low evenness, while the community where the species abundances are distributed equally within the community are considered as balance community.

Rarefaction curve was constructed based on the observed number of OTUs as function of number of sequences analyzed with QIIME to compare between non-infected and infected groups. Estimates of beta diversity were made using both unweighted UniFrac and weighted UniFrac (25) followed by principal coordinate analysis (PCoA) in QIIME to characterize the microbial population diversity. To analyze the relative abundance of the microbial members at the family level, we identified eight major family groups that adhered to two conditions: classified OTU and population density detectable at more than 2% of the total community in all samples. For OTUs that were unclassified or in low abundances (below 2%), were binned together in others/unknown category. The results from the QIIME were further analyzed with linear discriminate analysis effect size (LEfSe) (26). Then Kruskal–Wallis rank sum test was used to identify significantly differential abundance of the microbiota community between the comparison groups. Differentially distributed microbiome taxa were identified based on Ribosomal Database Project (RDP) that generated LEfSe cladograms for the each category comparison. Cladograms that had statistically significant taxonomic differences between the groups were identified. Significant alpha values of 0.05 and effect size threshold of 2 were used in the LEfSe analysis.

### Statistical Analysis

Splenic and cecal bacterial burden recovered from individual chickens between comparison groups were evaluated using unpaired *t*-test by the GraphPad Prism version 6.00 (GraphPad Software, La Jolla, CA, USA, www.graphpad.com). Furthermore, statistically significant differences in alpha diversity metrics were determined by performing Mann–Whitney *U* test with the Prism. Comparisons of relative abundance level of microbial members at the family level between different categories of comparisons that include treatment at different time point of experiment (non-infected at 3 and 8 D vs. *S*. Enteritidis-infected chickens at 2 and 7 DPI, respectively), days post infection (2 vs. 7 DPI), and age (3 vs. 8 D), were evaluated by performing Wilcoxon rank sum test with the JMP statistical software (version 12). To compare the relative abundance of dominant bacteria group at the family level in both non-infected and infected group, correlation coefficients and linear regression were also performed using the JMP.

## Results

### Effect of MHC Haplotypes on the Degree of Susceptibility to *S*. Enteritidis Infection Between Two Genetically Distinct Inbred Layer Lines

To determine whether the chicken MHC haplotype difference between the two genetic lines has an effect on the resistance or susceptibility to *S*. Enteritidis infection, kinetics of *S*. Enteritidis dissemination into spleen organ was examined to characterize the phenotypic difference between the two genetically distinct inbred lines. Three replicate trials were carried out. There was significant difference in splenic bacterial load detected between the two genetic lines at 2 DPI for the trial 1 (*p* < 0.0001) and combined data of three trials (*p* < 0.01) (Figure S1 in Supplementary Material). *S*. Enteritidis-infected chickens from UCD254 showing significantly higher bacterial burden at 2 DPI than from UCD077. There was no significant difference in splenic bacterial load at 7 DPI for either three individual trials or combined data of three trials.

The intestinal colonization level in the ceca of the two lines was also evaluated. There was no significant difference between two genetic lines in both trials 1 and 2 for both time points except trial 3 at 2 DPI (*p* < 0.0001). *S*. Enteritidis colonization level in cecal was significantly higher for UCD077 than UCD254 (Figure S2 in Supplementary Material). However, combined data from all three trials was significant at 7 DPI (*p* < 0.01) with higher cecal colonization detected in UCD254 than in UCD077 (Figure S2 in Supplementary Material).

### MHC Haplotype Effect on Microbiota Composition in Non-Infected and Infected Chickens

A total of 1,773,077 reads were generated from a total of 148 individuals combined from all three trials. Altogether, 15,618 different OTUs were identified from 50 non-infected chickens and 98 *S*. Enteritidis-infected chickens with 64 samples from 2 DPI and 34 samples from 7 DPI. There was no significant difference in alpha diversity metrics between two genetic lines of non-infected chickens at both 3 and 8 D (Figures S3 and S4 in Supplementary Material).

Alpha diversity metrics evaluated between two genetic lines of *S*. Enteritidis-infected chickens showed no significant difference at both days of postinfection periods (2 and 7 DPI) for all indices except for Simpson’s diversity in *S*. Enteritidis-infected groups at 7 DPI (*p* < 0.05) (Figures S5 and S6 in Supplementary Material).

### Developmental Differences in Cecal Microbiota Composition in Non-Infected Chickens

**Figure 1:**
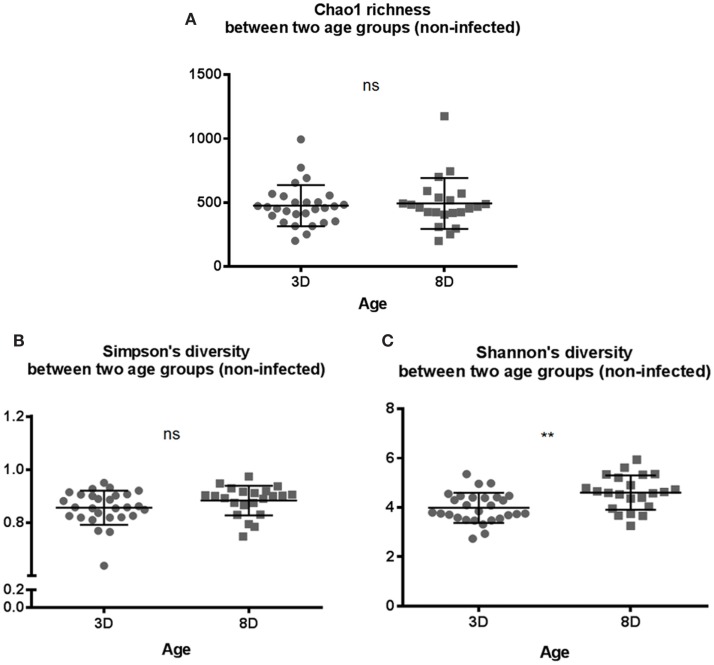
**Microbial alpha diversity between two age groups of 3 days old (3 D) and 8 days old (8 D)**. Alpha diversity metrics of **(A)** Chao1 richness estimate, **(B)** Simpson’s diversity, and **(C)** Shannon’s diversity index were analyzed. Shannon’s diversity index was significantly higher for 8-days-old chicks suggesting that as number of species increases, there is more even distribution of species in the community compared to 3-days-old chicks. All three diversity metrics were evaluated using Mann–Whitney *U* test. **p value < 0.01 and ns = non-significant.

In general, the results above suggested that there was no significant difference in microbial composition between two genetic lines. Therefore, data from both genetic lines were combined for further analysis. The alpha diversity metrics were compared between the two age groups of non-infected chickens. Both Chao1 richness and Simpson’s diversity showed no significant differences between the two age groups (Figures 1A,B). However, chickens at 8 days old of age had significantly more diverse microbial community structure in Shannon’s index (*p* < 0.01), suggesting a more balance distribution of the species in the community in older chickens than younger birds (Figure 1C).

**Figure 2:**
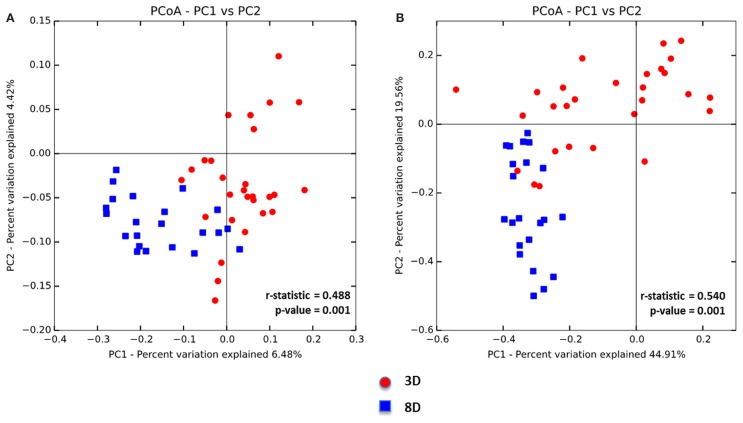
**Principal coordinate analysis (PCoA) based on (A) unweighted and (B) weighted UniFrac distances was analyzed for two age groups of non-infected chicks**. ANOISM with 999 permutations was used to detect the statistical significant difference between microbial communities of different groups, where both *r* and *p* value is reported. Abbreviations: 3 D, 3 days old; 8 D, 8 days old.

Beta diversity of the two age groups was also compared via unweighted UniFrac distance metric followed by the PCoA analysis (Figure 2A). Microbial composition differences between two age groups were significant (*p* = 0.001), but two clusters were not clearly separable (*r* = 0.488) (Figure 2A). Furthermore, weighted UniFrac distance metric was also used to compare between the two age groups followed by the PCoA analysis in considering the effect of relative abundance of microorganisms in each age group (Figure 2B). ANOSIM analysis showed significant difference in microbial community structure between two age groups (*p* = 0.001) and higher *r* value of more than 0.5 (*r* = 0.5403) indicated that separation of two groups was significant (Figure 2B).

**Figure 3:**
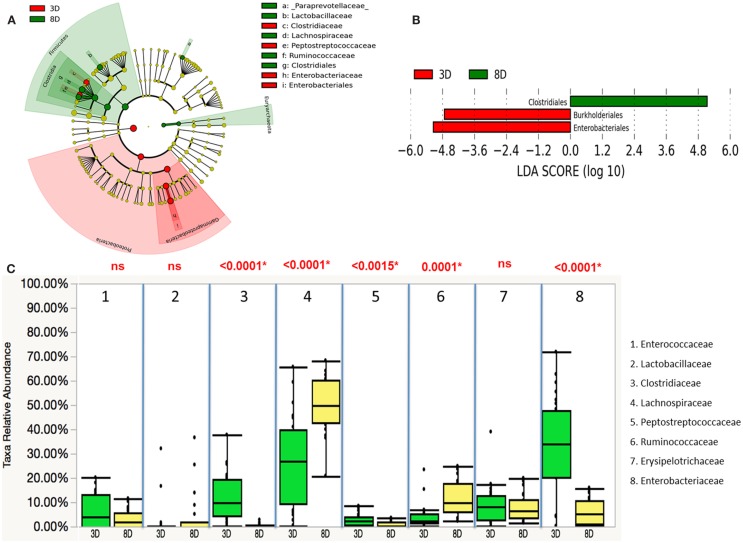
**Differential abundances of cecal microbial communities between two age groups**. **(A)** Taxonomic cladogram generated from LefSe analysis showing significant difference in microbiota profile of two age groups, red represented the enriched taxa in 3 days olds’ microbial community and green represented the enriched taxa in 8 days olds’ microbial community. **(B)** Differently abundant taxa detected with cut-off value of linear discriminant analysis (LDA) score >2.0. Enriched taxa in 8-days-old chicks (green) were indicated with positive LDA score, while taxa enriched in 3-days-old chicks (red) have negative LDA score. **(C)** Comparison of relative abundance levels of cecal microbiota at family level in 3 and 8 days old chicks was evaluated. The boxplot shows the quartiles above and below the median with dark line at center of the box denoting median, black dots showing the outlier. The respective *p* value for each family group is reported using Wilcoxon rank sum test. Abbreviations: 3 D, 3 days old; 8 D, 8 days old.

Microbiota compositions between two age groups were further analyzed using the linear discriminant analysis with effect size (LEfSe). Differentially abundant phyla detected in the age groups showed that Proteobacteria phylum was most dominantly present in younger chickens (3 D), while the most abundant phylum was Firmicutes for the older chickens (8 D) (Figure 3A). Three differentially represented core major groups at the order level were identified. For 8-day-old chickens, overrepresentation of Clostridiales and underrepresentation of Burkholderiales and Enterobacteriales were found (Figure 3B). Relative abundance of microbiota composition differences at eight major families were then compared between the two age groups using a Wilcoxon rank sum test. There was significantly marked decrease in *Clostridiaceae* (*p* < 0.0001), *Peptostreptococcaceae* (*p* < 0.0015), and *Enterobacteriaceae* (*p* < 0.0001) and higher abundance of *Lachnospiraceae* (*p* < 0.0001), and *Ruminococcaceae* (*p* < 0.0001) in older chickens than in young chickens (Figure 3C). In addition, the correlation between different members of the gut microbiota was also assessed for eight major families. A strong inverse correlation was observed between the *Enterobacteriaceae* and *Lachnospiraceae* (*r* = −0.7881, *p* < 0.0001), which suggested potential competition between these two members of the community.

### Developmental Differences in Cecal Microbiota Composition in Infected Chickens

**Figure 4:**
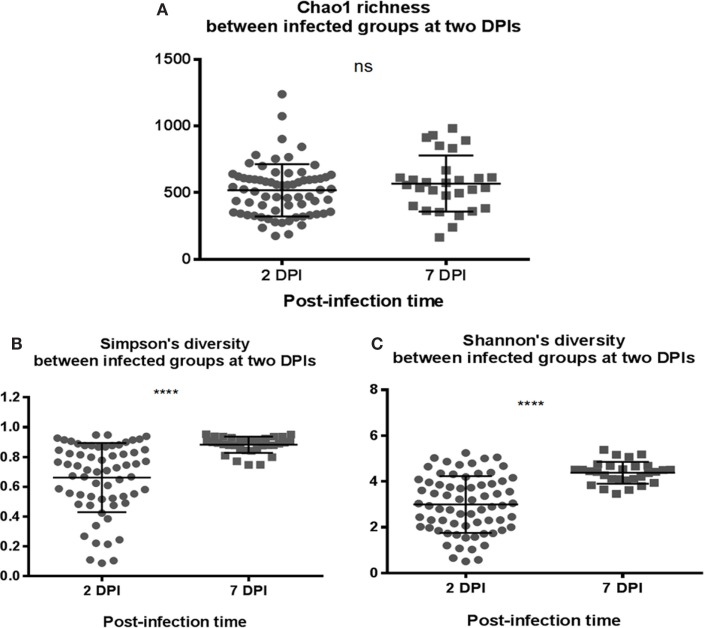
**Comparison of the diversity indices of *S*. Enteritidis-infected chicks at different time-points of post infection periods at 2 and 7 DPI**. **(A)** Chao1 richness estimate, **(B)** Simpson’s diversity, and **(C)** Shannon’s diversity index were analyzed. **(B,C)** Simpson and Shannon’s diversity showed significant difference in microbial diversity between two infected groups of chicks with increased diversity in microbiota composition at 7 DPI compared to 2 DPI. Both diversity metrics were evaluated using Mann–Whitney *U* test. ****p value < 0.0001.

**Figure 5:**
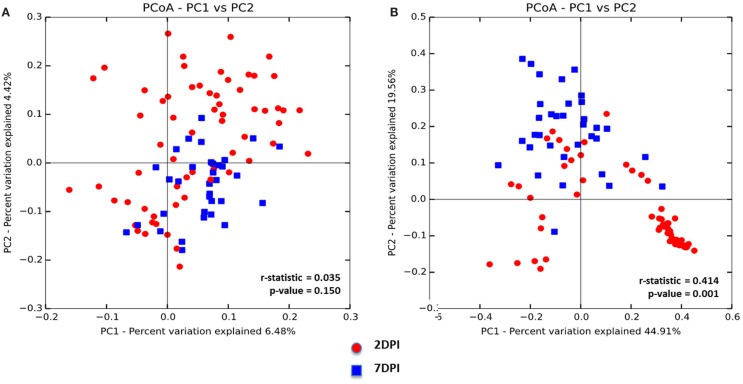
**Principal coordinate analysis (PCoA) based on (A) unweighted and (B) weighted UniFrac distances was analyzed for two age groups of infected chicks**. ANOISM with 999 permutations was used to detect the statistical significant difference between microbial communities of different groups, where both *r* and *p* value is reported. Abbreviations: 2 DPI, 2 days post infection; 7 DPI, 7 days post infection.

**Figure 6:**
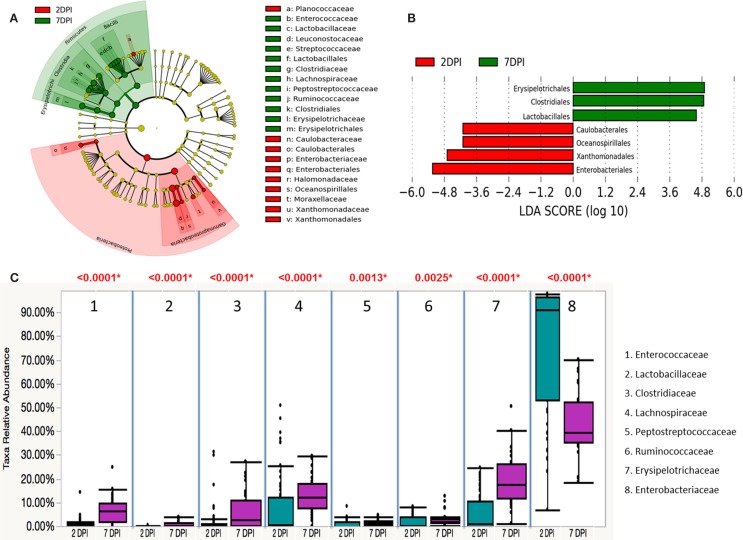
**Differential abundances of cecal microbial communities between two postinfection times of *S*. Enteritidis-infected chicks**. **(A)** Taxonomic cladogram generated from LefSe analysis showing significant difference in microbiota profile of 2 DPI (red) and 7 DPI (green). **(B)** Differently abundant taxa detected with cut-off value of linear discriminant analysis (LDA) score >2.0. Enriched taxa in 2 DPI group are indicated with negative LDA score (red) and taxa enriched in 7 DPI have positive LDA score (green). **(C)** Comparison of relative abundance levels of cecal microbiota at family level in *S*. Enteritidis-infected chicks between two postinfection times was evaluated. The boxplot shows the quartiles above and below the median with dark line at center of the box denoting median, black dots showing the outlier. The respective *p* value for each family group is reported using Wilcoxon rank sum test. Abbreviations: 2 DPI; 2 days post infection, 7 DPI; 7 days post infection.

To assess whether the microbiota diversity of the infected chickens differed between the two postinfection periods, both alpha and beta diversity indices were analyzed. There was no significant difference in Chao1 richness index (Figure 4A). However, both Simpson and Shannon indices showed highly significant difference in microbiota diversity (*p* < 0.0001) with increased diversity at 7 DPI compared to 2 DPI (Figures 4B,C). There was no significant difference in PCoA plot of unweighted UniFrac distance (*r* = 0.035, *p* = 0.150) (Figure 5A) between two postinfection time points. On the other hand, the weighted UniFrac distance analyzed with PCoA plot showed significant difference between two groups with *p* = 0.001 from ANOSIM analysis, suggesting the relative abundance of dominant taxa contributing to the differences, although the *r* value (*r* = 0.414) did not meet the cut-off threshold of 0.5 defined as two separated microbial community (Figure 5B). With LEfSe analysis, the phylum of Proteobacteria was dominated at (2 DPI) while Firmicutes phylum was found to be most abundant at 7 DPI (Figure 6A). At 7 DPI, a total of seven core microbiome groups at the order level were identified with enrichment of Erysipelotrichales, Clostridiales, and Lactobacillales (Figure 6B). Specifically at the family level, significantly (*p* < 0.0001) highly relative abundant of *Enterococcaceae*, *Lactobacillaceae*, *Clostridiaceae*, *Lachnospiraceae*, *Erysipelotrichaceae*, *Peptostreptococcaceae* (*p* = 0.0013), and *Ruminococcaceae* (*p* = 0.0025), and lower levels of *Enterobacteriaceae* (*p* < 0.0001) were observed at 7 DPI (Figure 6C). These findings indicated a slow recovery of microbial diversity in the infected individuals at 7 DPI with significant reduction of the dominant *Enterobacteriaceae* family.

**Figure 7:**
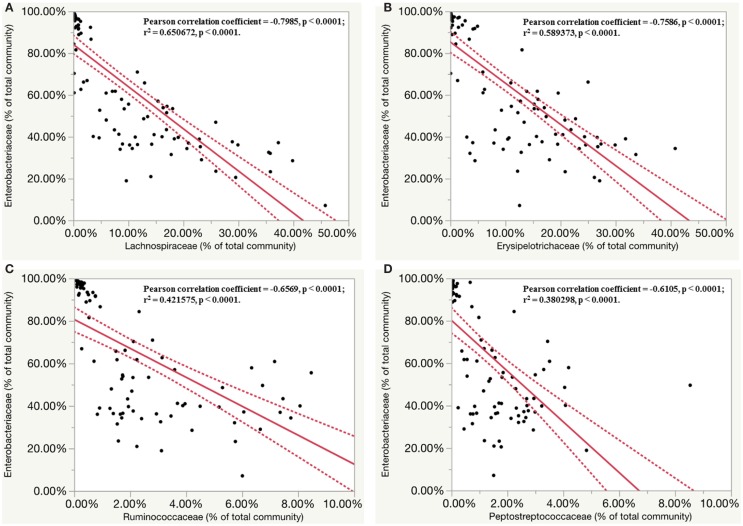
**Inverse correlation between relative abundance of members of *Enterobacteriaceae* family with other four major family groups**. Dashed line indicated 95% confidence intervals. **(A)** Linear regression plots of relative abundance of bacteria (% of total community) belonging to *Enterobacteriaceae* family and *Lachnospiraceae* family in *S*. Enteritidis-infected group. **(B)** Linear regression plots of relative abundance of bacteria belonging to *Enterobacteriaceae* family and *Erysipelotrichaceae* family in *S*. Enteritidis-infected group. **(C)** Linear regression plots of relative abundance of bacteria belonging to *Enterobacteriaceae* family and *Ruminococcaceae* family in *S*. Enteritidis-infected group. **(D)** Linear regression plots of relative abundance of bacteria belonging to *Enterobacteriaceae* family and *Peptostreptococcaceae* family in *S*. Enteritidis-infected group.

While assessing the microbiota profile of individual chick within the *S*. Enteritidis-infected group, a general trend pattern with an increase in *Enterobacteriaceae* accompanied by either a decrease or absence of *Lachnospiraceae* and *Ruminococcaceae* was found at the family classification level (Figures S7 and S8 in Supplementary Material). Therefore, the inverse correlations in relative abundance of *Enterobacteriaceae* with seven other major family groups were further evaluated. Significant inverse correlations (*p* < 0.0001) were found between *Enterobacteriaceae* and four other bacterial families including *Lachnospiraceae* (*r* = −0.7985), *Erysipelotrichaceae* (*r* = −0.7586), *Ruminococcaceae* (*r* = −0.6569) and *Peptostreptococcaceae* (*r* = −0.6105). Linear regression analysis revealed that the population density of *Enterobacteriaceae* was negatively correlated with other family members of the community (Figures 7A–D).

### *S*. Enteritidis-Associated Alteration in Chicken Cecum Microbiota Profile

**Figure 8:**
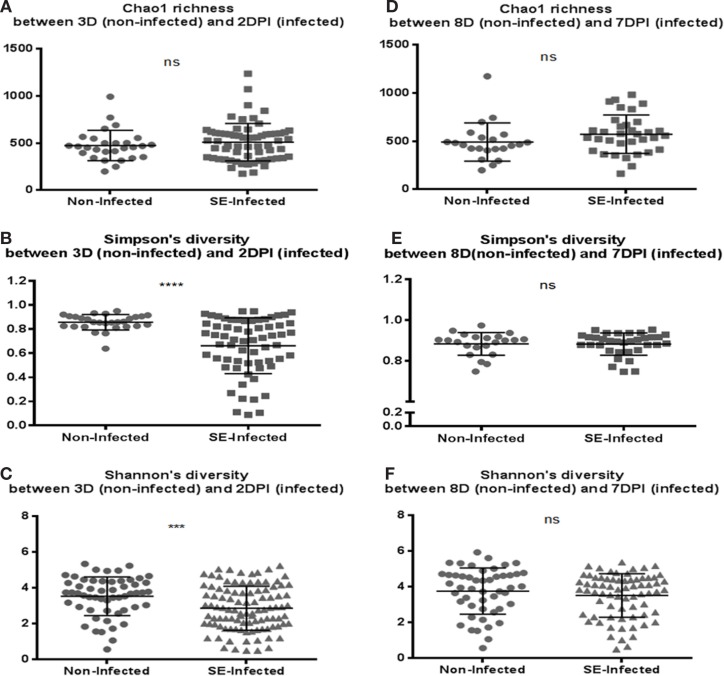
**Comparison of the diversity indices between the non-infected chicks and *S*. Enteritidis-infected chicks at 2 and 7 DPI: (A,D) Chao1 richness estimate, (B,E) Simpson’s diversity, and (C,F) Shannon’s diversity**. At 2 DPI, there was significant reduction in microbial diversity of the *S*. Enteritidis-infected chicks compared to non-infected chicks for measurement of both Simpson and Shannon’s diversity indices. Diversity metrics were evaluated using Mann–Whitney *U* test. ***p value <0.001, ****p value <0.0001, and ns = non-significant.

**Figure 9:**
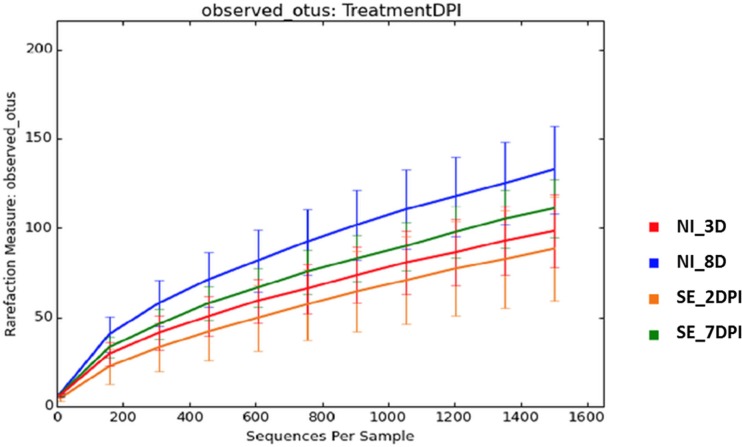
**Rarefaction curves of number of observed OTUs based on 97% sequence similarities for treatment group at different time points**. Abbreviations: NI_3 D, non-infected chicks at 3 days old; NI_8 D, non-infected chicks at 8 days old; SE_2 DPI, *S*. Enteritidis-infected chicks at 2 days post infection; SE_7 DPI, *S*. Enteritidis-infected chicks at 7 days post infection.

**Figure 10:**
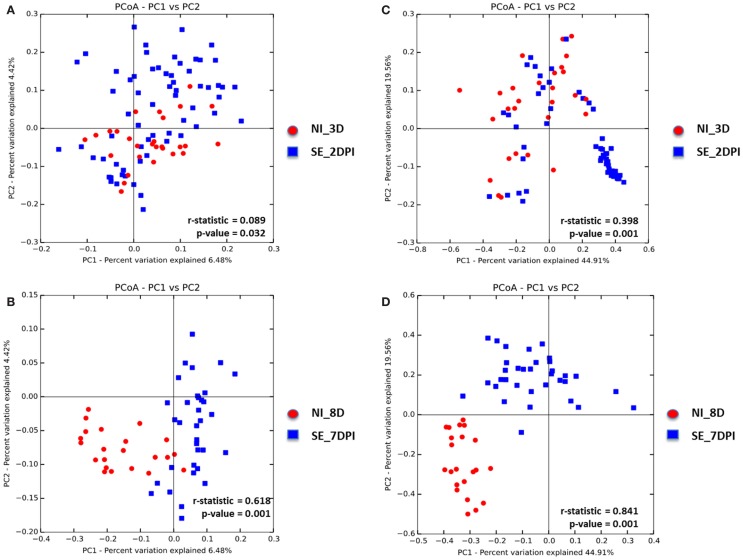
**Principal coordinate analysis (PCoA) based on (A,B) unweighted and (C,D) weighted UniFrac distances was analyzed for same-age group comparison between non-infected and infected chicks**. ANOISM with 999 permutations was used to detect the statistical significant difference between microbial communities of different groups, where both *r* and *p* value is reported. Abbreviations: NI_3 D, non-infected chicks at 3 days old; SE_2 DPI, *S*. Enteritidis-infected chicks at 2 days post infection; NI_8 D, non-infected chicks at 8 days old; SE_7 DPI, *S*. Enteritidis-infected chicks at 7 days post infection.

Microbiota data were also analyzed to examine the effect of S. Enteritidis infection at two different postinfection times of the experiments. With *S*. Enteritidis infection, Chao1 richness showed no differences between the two groups at both postinfection time points (Figures 8A,D). Both Simpson and Shannon’s diversity indices showed that there was significant reduction in microbiota diversity of the *S*. Enteritidis-infected chickens at 2 DPI compared to non-infected chickens (*p* < 0.0001 and *p* < 0.001, respectively) (Figures 8B,C). However, there was no significant difference between non-infected and *S*. Enteritidis-infected groups at 7 DPI for both indices (Figures 8E,F). Rarefaction curves highlighted a lower species richness of *S*. Enteritidis-infected groups at both time points compared to non-infected groups (Figure 9). Beta diversity was also analyzed to examine differences or similarities in cecal microbiota community composition between non-infected and *S*. Enteritidis-infected groups. PCoA plots based on unweighted UniFrac distance metric showed that there was significant separation in microbial community of non-infected and infected chickens at later postinfection time of 7 DPI (*p* = 0.001, *r* = 0.618) compared to early postinfection time at 2 DPI (*p* = 0.032, *r* = 0.089) (Figures 10A,B). With the PCoA plot based on weighted UniFrac distance metric where the relative abundance of OTUs were considered, there was more significant clustering pattern observed between the 8-day-old non-infected chickens and the same-age infected counterparts at 7 DPI (*p* = 0.001, *r* = 0.841) (Figure 10D). In contrast, the microbial communities of non-infected and infected groups at early time points (3 D vs. 2 DPI) showed no visible separation between two groups (*r* = 0.398) although the *p* value = 0.001 (Figure 10C).

To compare OTUs abundance between two treatment groups that were significantly different, ANOVA test was performed. Abundance of *Lachnospiraceae* family was found to significantly decrease in *S*. Enteritidis-infected group compared to the non-infected group [false discovery rate (FDR) < 0.05]. GI tract of young layer chickens were dominated by two main phyla belonging to Firmicutes and Proteobacteria. With *S*. Enteritidis infection, major phylum level shifted toward increased abundance of Proteobacteria at both time points when compared to non-infected same-age counterpart (Figures S9 and S10 in Supplementary Material). Representative of the bacterial family belonging to *Enterobacteriaceae*, *Erysipelotrichaceae*, *Ruminococcaceae*, *Peptostreptococcaceae*, *Lachnospiraceae*, *Clostridiaceae*, *Lactobacillaceae*, and *Enterococcaceae* dominated in the cecal microbiota of the young layer chickens.

**Figure 11:**
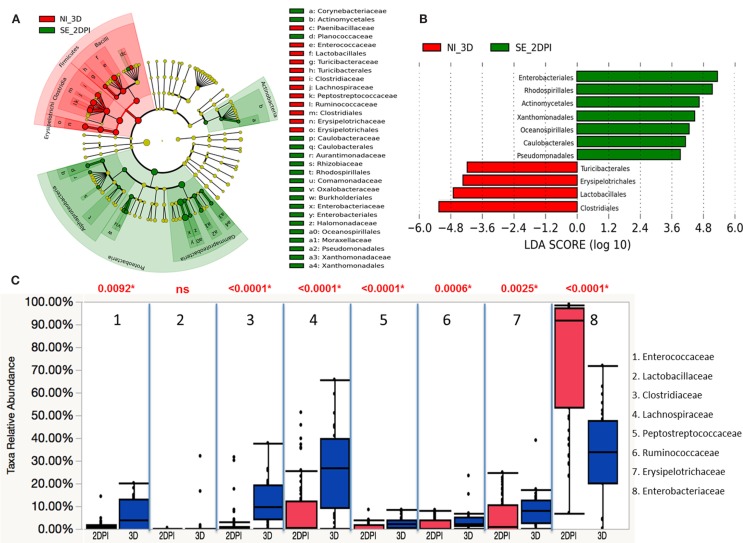
**Differential abundances of cecal microbial communities between infected and non-infected group at 2 DPI**. **(A)** Taxonomic cladogram generated from LefSe analysis showing significant difference in microbiota profile of non-infected (red) and *S*. Enteritidis-infected (green) at early days post infection (2 DPI). **(B)** Differently abundant taxa detected with cut-off value of linear discriminant analysis (LDA) score >2.0. Non-infected enriched taxa are indicated with negative LDA score (red) and taxa enriched in *S*. Enteritidis-infected have positive LDA score (green). **(C)** Comparison of relative abundance levels of cecal microbiota at family level in treatment group was evaluated. The boxplot shows the quartiles above and below the median with dark line at center of the box denoting median, black dots showing the outlier. The respective *p* value for each family group is reported using Wilcoxon rank sum test. Abbreviation: NI_3 D, non-infected chicks at 3 days old; SE_2 DPI, *S*. Enteritidis-infected chicks at 2 days post infection.

The microbiota compositions of non-infected were compared against the same-age counterparts of chickens in infected groups with LEfSe. The phyla of Actinobacteria and Proteobacteria were significantly enriched in the *S*. Enteritidis-infected groups of 2 DPI, while Firmicutes were significantly enriched in the non-infected group of 3 D (Figure 11A). Differentially representation of 11 groups at order level were identified with underrepresentation of four groups and enrichment of seven groups in the *S*. Enteritidis-infected group at 2 DPI (Figure 11B). Using a Wilcoxon rank sum test by JMP software, the relative abundance of gut microbes at the family level was compared between the *S*. Enteritidis-infected group of 2 DPI and the non-infected group of 3 D (Figure 11C). A marked decrease in *Enterococcaceae* (*p* = 0.0092), *Clostridiaceae* (*p* < 0.0001), *Lachnospiraceae* (*p* < 0.0001), *Peptostreptococcaceae* (*p* < 0.0001), *Ruminococcaceae* (*p* = 0.0006), and *Erysipelotrichaceae* (*p* = 0.0025) was found in the *S*. Enteritidis-infected group. On the other hand, *Enterobacteriaceae* (*p* < 0.0001) were highly abundant in the *S*. Enteritidis-infected group at 2 DPI.

**Figure 12:**
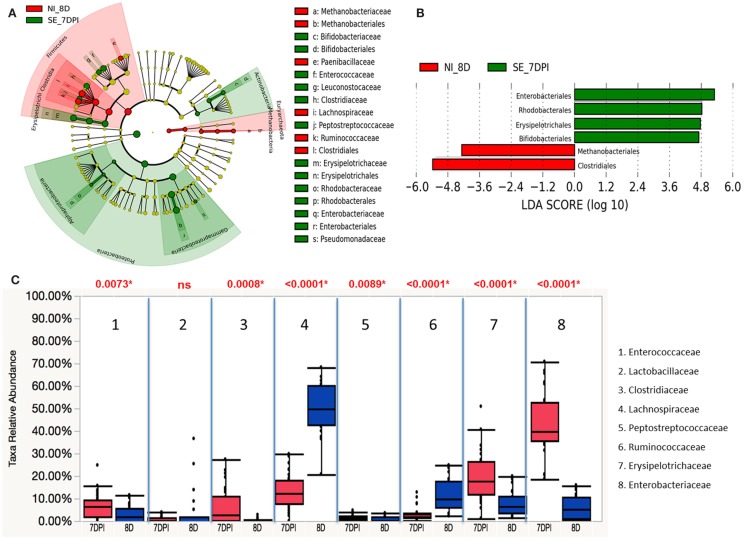
**Different abundances of cecal microbial communities between infected and non-infected group at 7 DPI. (A) Taxonomic cladogram generated from LefSe analysis showing significant difference in microbiota profile of non-infected (red) and *S*. Enteritidis-infected (green) at 7 DPI**. **(B)** Differently abundant taxa detected with cut-off value of linear discriminant analysis (LDA) score >2.0. Non-infected enriched taxa are indicated with negative LDA score (red) and taxa enriched in *S*. Enteritidis-infected have positive LDA score (green). **(C)** Comparison of relative abundance levels of cecal microbiota at family level in treatment group was evaluated. The boxplot shows the quartiles above and below the median with dark line at center of the box denoting median, black dots showing the outlier. Abbreviation: NI_8 D, non-infected chicks at 8 days old; SE_7 DPI, *S*. Enteritidis-infected chicks at 7 days post infection.

Similarly, both Actinobacteria and Proteobacteria phyla were also enriched in *S*. Enteritidis-infected groups of 7 DPI, while the non-infected group at 8 D showed significant abundance in Firmicutes and Euryarchaeota phyla (Figure 12A). A total of six groups at order level were differentially represented with underrepresentation of two groups and overrepresentation of four groups in *S*. Enteritidis-infected group at 7 DPI (Figure 12B). Using Wilcoxon rank sum test, higher abundance level of *Enterococcaceae* (*p* = 0.0073), *Clostridiaceae* (*p* = 0.0008), *Peptostreptococcaceae* (*p* = 0.0089), *Erysipelotrichaceae* (*p* < 0.0001), and *Enterobacteriaceae* (*p* < 0.0001) were found in the *S*. Enteritidis-infected group than in the non-infected group at 7 DPI. In contrast, *Lachnospiraceae* (*p* < 0.0001), and *Ruminococcaceae* (*p* < 0.0001) were significantly decreased in the infected group compared to the non-infected same-age counterparts (Figure 12C).

## Discussion

Maintenance of intestinal microbiota homeostasis is a key determinant for overall health and nutrition state of the host. Development of gut microbiota in the chick begins immediately after the hatch where a number of external factors such as environment, feed, and contact with chick handler could influence the overall microbial community structure (27). In addition, the host genotype is another important factor in affecting the composition of the gut microbiota (7, 9, 11). Early encounter with diverse enteric pathogens present in the environment during posthatch period also poses great risk for newly hatched chicks. This early host–pathogen interaction could potentially impact the further colonization of other microbes and shape the overall structure of gut microbiota. In this study, we therefore address this dominant potential of early pathogen exposure on shaping the microbiota composition by using 1-day-old chickens from two genetically distinct lines as infection model.

Chicken MHC and its association with resistance to avian disease, such as Marek’s disease, infectious bronchitis virus (IBV), avian influenza virus, ectoparasite, and *Staphylococcus aureus* had been documented in several studies (14, 15, 17, 20). However, the role of MHC on the resistance to *S*. Enteritidis infection had been contradictory. Study by Cotter et al. (13) suggested an association of MHC B haplotype with resistance to *S*. Enteritidis infection in a neonatal chick infection model of 12 MHC-congenic chicken lines. On the other hand, another study by the Bumstead and Barrow (12) stated that there was no evidence to support strong association of MHC haplotypes with resistance to *Salmonella typhimurium* in newly hatched chicks. Our findings in the current study indicated that MHC haplotype had significant effects only on early stage of systemic infection of *S*. Enteritidis (splenic bacterial burden difference at 2 DPI) and on late stage of local infection of *S*. Enteritidis (cecal bacterial burden difference at 7 DPI). However, in general, MHC genetic background had a limited effect on resistance to *S*. Enteritidis infection.

Developmental stage can have a significant impact on the microbiota composition in the GI tract. The early inoculum challenged to young chickens in this study could be a possible driving force in potentially rendering the microbiota composition of chickens of late stage. Without pathogen infection, microbiota diversity and complexity often increase with age of the chick (28, 29). Temporal changes in the chicken gut microbiota with aging could have important consequences on susceptibility to pathogen infection. The use of very young chickens as infection model is important for the current study as chickens are often exposed to *Salmonella* at very young age in natural setting. However, the immaturity of the immune system as well as non-establishment of complex microbiota in young chickens could have a significant effect on the outcome of the infection (30). Beaumont et al. (31) showed that increased resistance to *Salmonella* at adult chickens was negatively correlated to genetic resistance at the young age. Therefore, MHC haplotype effect on microbiota profile could be different by the use of different age infection model. Although it is beyond the scope of the current study, challenging at 2 weeks of age instead of 1-day-old chicks could provide additional insights of host genetic background impact on microflora composition and warranted further investigation.

In the non-infected chickens, Firmicutes followed by Proteobacteria phylum dominated the microbiota composition. Temporal fluctuation in the microbial community structure at the family level was observed as chick aged (from 3 to 8 D). *Enterobacteriaceae* family was significantly enriched in younger chickens, while *Lachnospiraceae* and *Ruminococcaceae* families were more abundant with a reduction in *Enterobacteriaceae* family in older chickens. The overall bacterial diversity in early life stage of chick host (both age groups) in the current study was low with a few members predominantly occupying the GI tract, which was in agreement with other studies (28, 29). Similar to another study, the non-infected chickens at 3 days old had high abundance of *Enterobacteriaceae* (32). Members of the *Enterobacteriaceae* family including bacterial pathogens like *Salmonella*, *Escherichia coli*, and *Shigella* are known enteric pathogens in the GI tract. Harboring high level of *Enterobacteriaceae* in young chickens could potentially increase their susceptibility to infection by related enteric pathogens. Indeed, studies in mouse model have shown that increased susceptibility to related enteric pathogen infections were observed in host whose microbiota composition was dominated by the presence of *E. coli* belonging to *Enterobacteriaceae* family (33, 34). The concept of “like will to like” was proposed by the Stecher et al. to help explain the bloom of closely related bacterial species in the GI tract that result in dysbiosis of microbiota in the disease host (33). It had been suggested that high prevalence of certain bacterial species in the microbiota community could alter the conditions within the gut that selectively confer the fitness advantage upon other related species within the same phylogenetic group (35, 36). We speculate that early colonization by members of *Enterobacteriaceae* family in GI tract of the newly hatched chickens could potentially precondition the intestinal tract of the chick host to allow easy colonization by enteric pathogens. Thus, depletion of certain bacteria taxa from *Enterobacteriaceae* family during the early posthatch period could potentially enhance the host resistance to enteric pathogen infection. This may be how growth promoters work in poultry feed as antibiotics in the growth promoters can eliminate certain members of *Enterobacteriaceae* family.

Once successful invasion by pathogen like *S*. Enteritidis is established within the niche of the GI tract, pathogen-associated alteration in microbial community structure occurred (37–39). Our results revealed that *S*. Enteritidis infection resulted in significant reduction in bacterial diversity specifically at early postinfection period. Reduction in bacterial diversity in the infected birds was partially attributed by the presence of the *Enterobacteriaceae* family that dominated the microbial community. Major phylum shift was observed in infected group at 2 DPI where there was expansion of Proteobacteria with concomitant reduction in Firmicutes phyla. This sudden shift in microbial population structure due to *S*. Enteritidis infection changed the ratio of two major phyla groups, which is the hallmark indicator of intestinal microbiota dysbiosis in disease host (40, 41). *Salmonella* associated alteration of the gut microbiota could be a result of either pathogen-commensal microbiota interaction or host mucosal immune response to the pathogen or even a combination of both (38). Host-mediated inflammation response triggered by the presence of the pathogen could also change the conditions within the GI tract to favor and support the growth of specific member of the microorganisms. Studies in mouse colitis models have showed that inflammation allow facultative anaerobes like *Salmonella* or other members of the *Enterobacteriaceae* family to utilize anaerobic respiration as alternative growth pathway to gain competitive advantage over resident microbes that are mostly obligate anaerobe (34, 38, 40, 42–44). The underlying mechanism that is driving the bloom of Proteobacteria phylum in chick host following *Salmonella* infection is not yet known. Whether similar route of respiration pathway is being utilized by *Salmonella* to gain growth advantage in inflamed chicken gut is the hypothesis that we are currently investigating.

Comparisons between microbial communities of non-infected and infected groups showed that the community structure of the two groups appeared to be more similar initially at an early development stage (3 D vs. 2 DPI). However, as time progresses with *S*. Enteritidis infection, a significant difference in community structure between the two groups was apparent, with clear separation in the group’s clustering pattern on PCoA plots (8 D vs. 7 DPI). This result suggests that the impact of *S*. Enteritidis infection on microbial communities was more substantial in late stage than in early stage. A study by Videnska et al. (45) found that members of the families *Lachnospiraceae* and *Ruminococcaceae* are predominantly present in the 2-week-old laying chickens, and likely play an important role in the overall development of the gut microbial community. Further analysis at the family level found that two core members of the gut microbiota belonging to *Lachnospiraceae* and *Ruminococcaceae* families were significantly reduced in the infected groups. Our findings suggested that with *S*. Enteritidis infection, selective reduction of these bacterial genera could negatively impact gut microbial diversity and development. Although long-term impact of *S*. Enteritidis infection on microbiome development in adult chickens was not possible to be evaluated in the current study, further investigation in this regard could provide important insights on it.

Interestingly, a strong inverse correlation between *Enterobacteriaceae* and *Lachnospiraceae* was observed in both the non-infected and infected birds, suggesting a possible antagonistic interaction between the two members of these taxa that could influence the prevalence of different microbial populations in the gut. In addition, the abundance of members belonging to *Lachnospiraceae* family was significantly decreased with *S*. Enteritidis infection. Contrary to our findings, Videnska et al. (37) observed only minor modification in chicken gut microbiota with no significant changes in *Lachnospiraceae* family following *S*. Enteritidis infection. The discrepancies observed between this study and our findings may be attributed to different age of infection model, samples collected on different days of postinfection and different genetic background of chickens. *Lachnospiraceae* as well as another family, *Ruminococcaceae*, that also show significant reduction in the infected group, belong to the Clostridium clusters IV and XIVa (45). Members of these groups generate butyric acid, short chain fatty acids (SCFAs) that are produced as end products of fermentation of carbohydrate by anaerobic intestinal microbes. There is complex interplay between diet, SCFAs concentration, and microbiota composition that regulate the colonization level of members of the Proteobacteria phylum (36, 46). Depending on the type of SCFAs being produced and its concentration level in the gut, it can affect different members of microbial community in a different way. Specifically for *Salmonella*, high concentration of acetate production was found to increase the invasion gene expression of *Salmonella* Pathogenicity Island 1 (SPI1) (47). High concentration level of butyric acid, on the other hand, down-regulated the SPI1 gene expression level, which can reduce invasion capability of bacteria in the host (48). In the poultry industry, addition of butyric acid in feed has been shown to reduce both colonization and shedding of *Salmonella* in chickens (49, 50). Taken together, reduction in butyric acid producing bacteria such as *Lachnospiraceae* and *Ruminococcaceae* families with *S*. Enteritidis infection may implied that both producers and its products may have a potential protective role in providing colonization resistance against *Salmonella* infection or reducing the members of *Enterobacteriaceae* family in gut microbiota to maintain homeostasis. A novel *Salmonella* preventive strategies that implement combined approach of competitive exclusion bacteria with SCFAs should be explored to eliminate enteric pathogens and improve overall gut health of the chicken host.

In conclusion, our findings indicated that early exposure in young chickens to *Salmonella* influences and shape the overall microbiota composition. Microbial diversity was significantly reduced in *S*. Enteritidis-infected host compared to same-age non-infected group. Overall perturbation of microbiota community was found to be associated with expansion of *Enterobacteriaceae* family at early postinfection period. Decrease in butyrate producing bacteria belonging to *Lachnospiraceae* family was found to have a negative correlation with high prevalence of *Enterobacteriaceae* family, suggesting possible competitive interaction between the two bacterial taxa in the gut. Additionally, increased susceptibility to *Salmonella* infection in young chickens could be contributed by highly relative abundance of *Enterobacteriaceae* family in the gut. Predominance of this bacterial taxa could potentially confer competitive growth advantage upon its related species over resident microbiota during enteric infection via altering the environmental conditions of the GI tract of the host, which further promote the imbalance state of the young chick’s gut microbiota. This study provided a preliminary insight into the contributing role of early host–pathogen interaction that influences the composition makeup of gut microbiota.

## Author Contributions

KM performed the experiment, analyzed the sequencing data, and drafted the manuscript. HZ designed the experiment, provided the concept of the analysis, and was involved in critical revision of the manuscript. PS and MH ran the scripts for sequencing data and help revised the manuscript. GC and HC helped with animal trials, sample collection and DNA extraction of samples. LG and EM provided primers, contributed ideas for analyzing the data and helped in revising the manuscript. All authors submitted comments, read and approved the final manuscript.

## Conflict of Interest Statement

The authors declare that the research was conducted in the absence of any commercial or financial relationships that could be construed as a potential conflict of interest.
