# Supplementary material for: Salmonella enterica Serovars Enteritidis Infection Alters the Indigenous Microbiota Diversity in Young Layer Chicks
Source: Front Vet Sci. 2015 Nov 23;2:61. doi: 10.3389/fvets.2015.00061 (PMC4672283; doi:10.3389/fvets.2015.00061)
Supplement: Supplementary file 1 [file Data_Sheet_1.DOCX]

Supplementary Material

***Salmonella enterica* serovars Enteritidis infection alters the indigenous microbiota diversity in young layer chicks**

Khin K.Z. Mon, Perot Saelao, Michelle Halstead, Ganrea Chanthavixay, Huai-Chen Chang, Lydia Garas, Elizabeth A. Maga and Huaijun Zhou^*^

*** Correspondence:** Dr. Huaijun Zhou, [hzhou@ucdavis.edu](mailto:hzhou@ucdavis.edu)

# Supplementary Figures


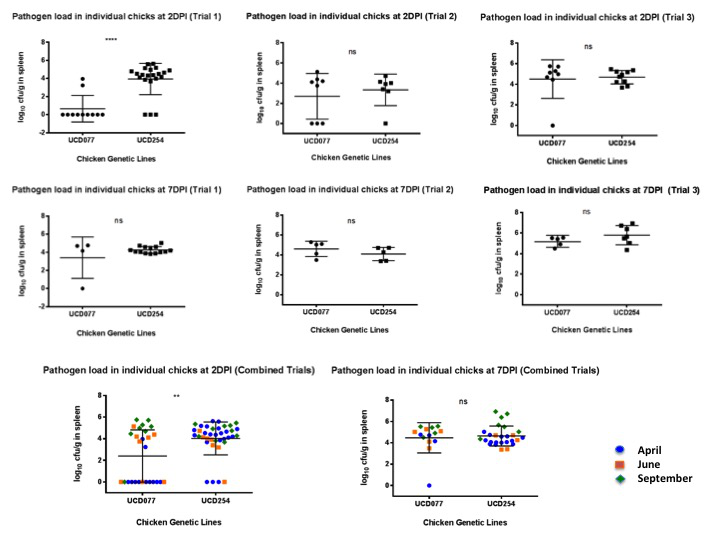


Figure S1: Splenic bacterial load recovered from *S.* Enteritidis-infected chicks between two genetic lines from each replicate trials as well as combined trials data. Data are presented in log10 cfu/g with each data points representing individual chick. Statistically significant differences between two lines were determined by unpaired t-test. ****p < 0.0001, **p < 0.01. ns = non-significant.


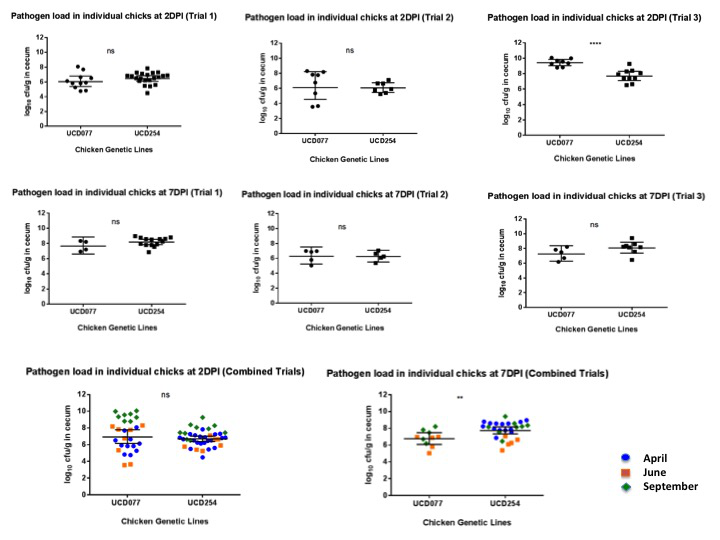


Figure S2: Cecal bacterial load recovered from *S.* Enteritidis-infected chicks between two genetic lines from each replicate trials as well as combined trials data. Data are presented in log10 cfu/g with each data points representing individual chick. Statistically significant differences between two lines were determined by unpaired t-test. **p < 0.01, ns = non-significant.


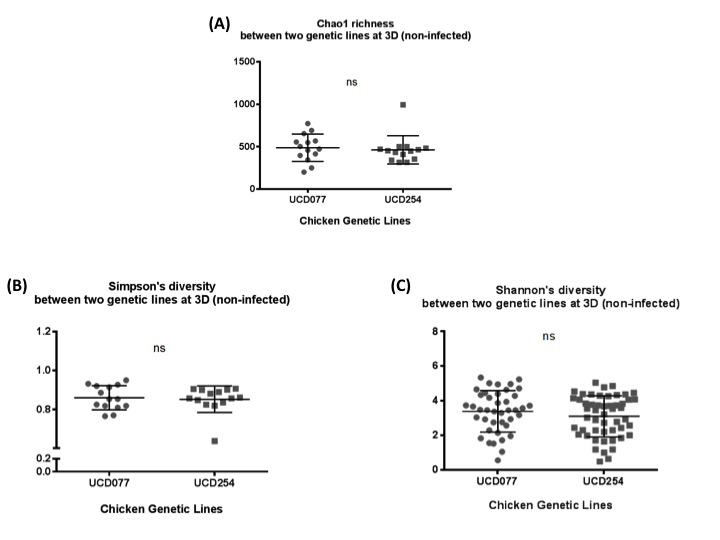


Figure S3: Microbial alpha diversity between genetic lines at 3 days old (3D). Alpha diversity metrics of **(A)** Chao1 richness estimate, **(B)** Simpson’s diversity, and **(C)** Shannon’s diversity index were analyzed. All three diversity metrics were evaluated using Mann-Whitney U test. ns = non-significant.


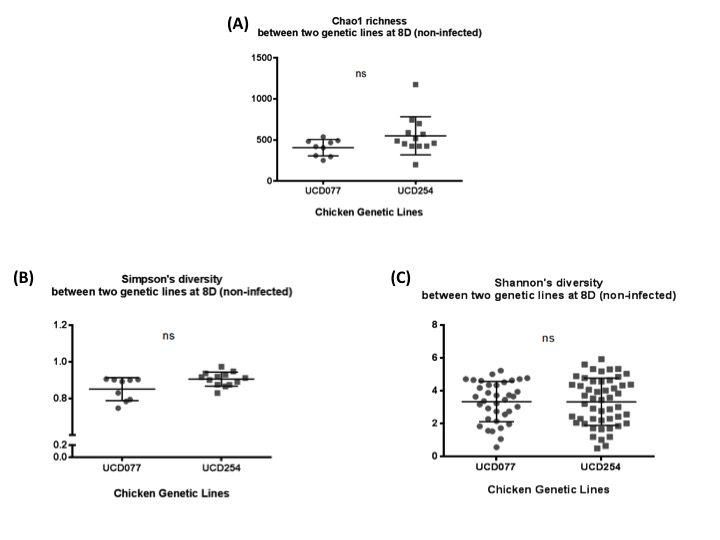


Figure S4: Microbial alpha diversity between two genetic lines at 8 days old (8D). Alpha diversity metrics of **(A)** Chao1 richness estimate, **(B)** Simpson’s diversity, and **(C)** Shannon’s diversity index were analyzed. All three diversity metrics were evaluated using Mann-Whitney U test. ns = non-significant.


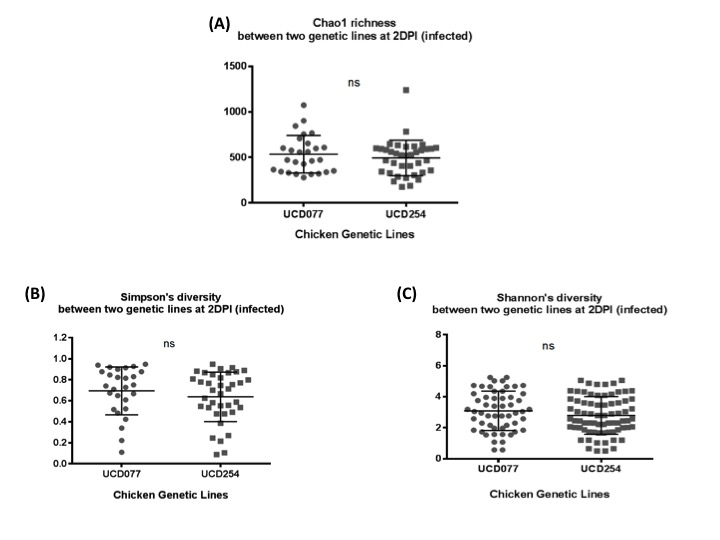


Figure S5: Microbial alpha diversity between two genetic lines at 2dpi of infected chicks. Alpha diversity metrics of **(A)** Chao1 richness estimate, **(B)** Simpson’s diversity, and **(C)** Shannon’s diversity index were analyzed. All three diversity metrics were evaluated using Mann-Whitney U test. ns = non-significant.


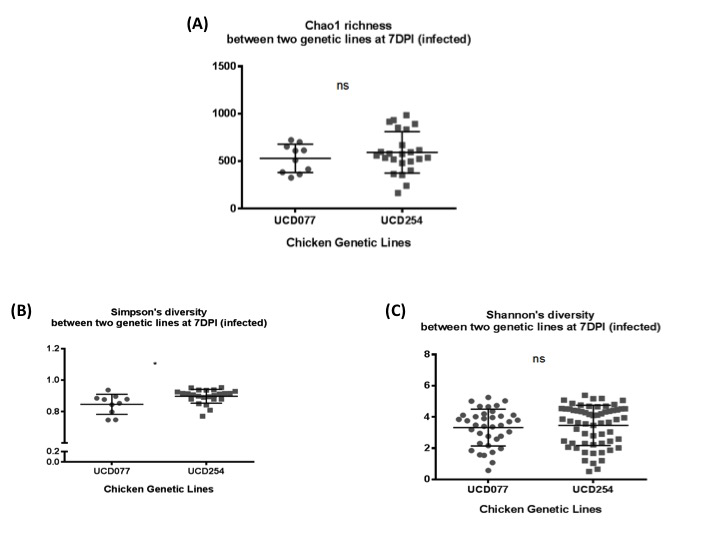


Figure S6: Microbial alpha diversity between two genetic lines at 7dpi of infected chicks. Alpha diversity metrics of **(A)** Chao1 richness estimate, **(B)** Simpson’s diversity, and **(C)** Shannon’s diversity index were analyzed. **(C)** Simpson’s diversity showed significant differences at 7dpi with increased microbial diversity for UCD254 line. All three diversity metrics were evaluated using Mann-Whitney U test. *p < 0.05 and ns = non-significant.


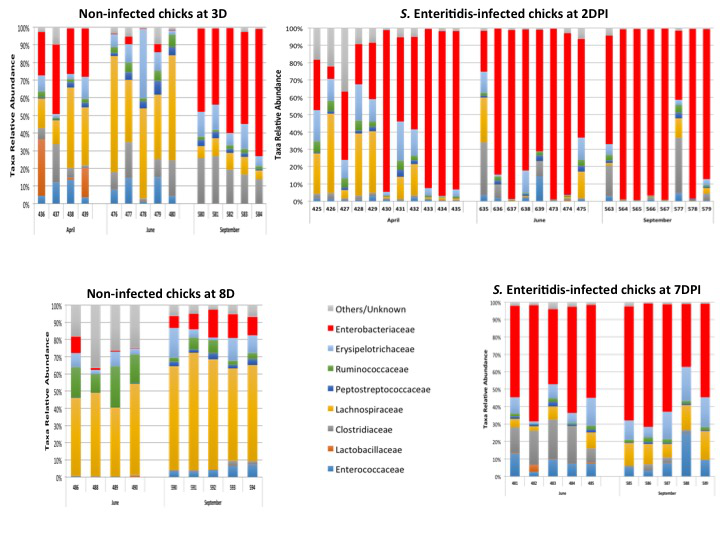


Figure S7: Relative abundance of major bacteria group at the family level in individual chicks based on treatment group (non-infected and infected) for UCD077 line across three replicate trials.


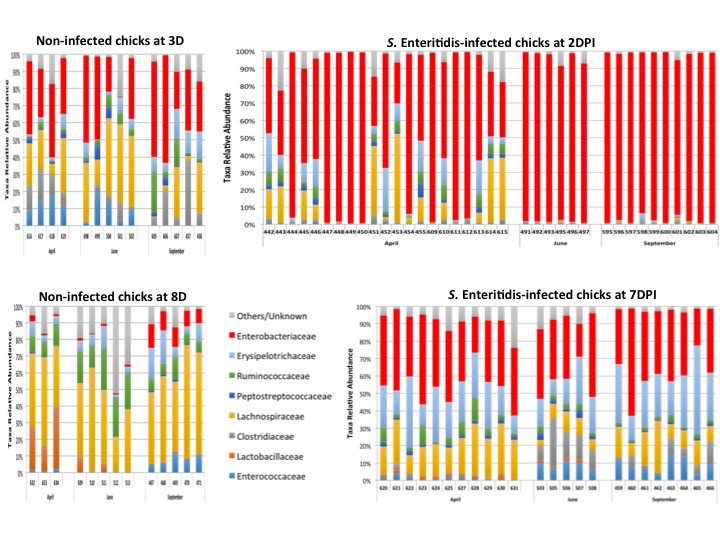


Figure S8: Relative abundance of major bacteria group at the family level in individual chicks based on treatment group (non-infected and infected) for UCD254 line across three replicate trials.


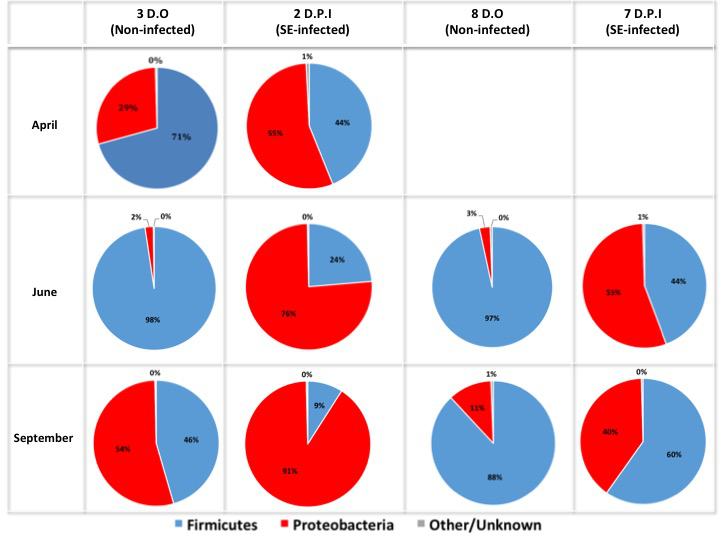


Figure S9: Pie chart representation of the relative abundances of major phyla detected for each groups of chicks from UCD077 across three replicate trials. Percentage representing each phylum of the total community.


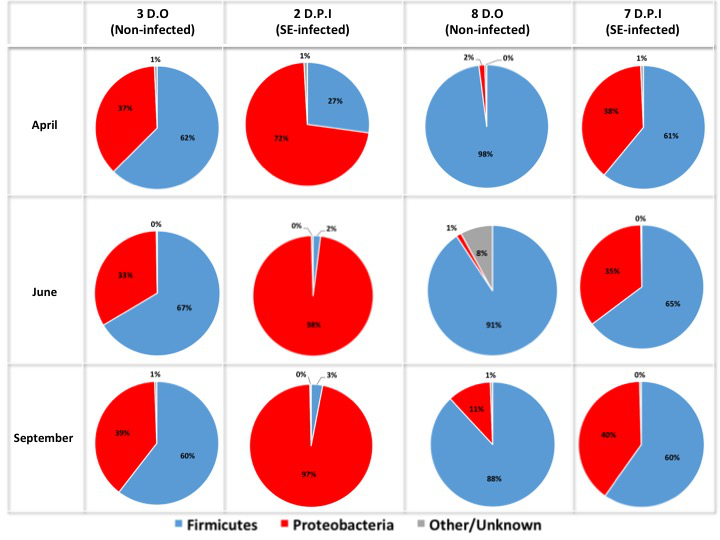


Figure S10: Pie chart representation of the relative abundances of major phyla detected for each groups of chicks from UCD254 line across three replicate trials. Percentage representing each phylum of the total community.
